# Stellate ganglion intervention for chronic pain: A review

**DOI:** 10.1002/ibra.12047

**Published:** 2022-05-28

**Authors:** Qingyang Luo, Song Wen, Xinran Tan, Xi Yi, Song Cao

**Affiliations:** ^1^ Department of Anesthesiology Affiliated Hospital of Zunyi Medical University Zunyi Guizhou China; ^2^ Department of Pain Medicine Affiliated Hospital of Zunyi Medical University Zunyi Guizhou China; ^3^ Guizhou Key Laboratory of Anesthesia and Organ Protection Zunyi Medical University Zunyi Guizhou China

**Keywords:** chronic pain, pulse radiofrequency, stellate ganglion, stellate ganglion block

## Abstract

Stellate ganglion (SG) intervention is currently widely being studied in many kinds of chronic pain. As one of the convenient ways to treat the sympathetic nervous system, the indications for stellate ganglion intervention (SGI) include complex regional pain syndrome, postherpetic neuralgia, cancer pain of different origins, orofacial pain, and so forth. SGI refers to the reversible or irreversible blocking of the cervical sympathetic trunk, cervical sympathetic ganglion, and their innervation range through noninvasive or minimally invasive treatment. Current treatment options include stellate ganglion block (SGB), SG pulsed radiofrequency, continuous radiofrequency treatment, and noninvasive SGB. In particular, SGB continues to be one of the most studied methods in chronic pain management. However, a single SGB usually provides only short‐term effects; repeated SGB may result in complications such as hoarseness, light‐headedness, and vessel or nerve injury. Meanwhile, the mechanism of SGI is still unclear. This review discusses the research progress of SGI methods, effectiveness, complications, and possible mechanisms in the management of chronic pain.

## Graphical abstract

The possible mechanism of stellate ganglion intervention (SGI) may relieve chronic pain associated with sympathetic maintenance by inhibiting dorsal root ganglion (DRG) nerve sprouting, norepinephrine release, and pain signaling generation in the DRG. Besides, SGI can relieve chronic pain, anxiety, and depression by inhibiting neuroinflammation in the spinal cord and in the brain.

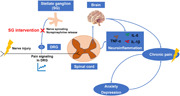

## 1 INTRODUCTION

Chronic pain is defined as pain that persists or recurs for more than 3 months, usually accompanied by negative emotions including anxiety and/or depression.^1^ Chronic pain severely affects not only physical and mental health but also places a huge economic burden on the family and society. The incidence of chronic pain is reported to be over 30% in China.^2^ Current conventional methods for the treatment of chronic pain include drug therapy, minimally invasive therapy, psychotherapy, and physical therapy.^3^ However, research has found that sympathetic nerves are involved in the maintenance of many kinds of chronic pain.^4^ Therefore, inhibition of sympathetic hyperexcitability may improve the therapeutic effect, which may be related to the involvement of sympathetic nerves in regulating glial inflammation.^5^ Among them, intervention of stellate ganglion (SG) is effective for pain in the head, face, neck, upper limbs, or upper chest. In this review, we summarize the clinical evidence available for the effectiveness of stellate ganglion intervention (SGI), and demonstrate clinical applications of SGI.

## 2 SG ANATOMY

SG is part of the sympathetic ganglion of the neck, formed by the merging of the inferior cervical ganglion and the first thoracic sympathetic ganglion in about 80% of the population, so the SG is also called the cervicothoracic ganglion.^6^ SG is located anteriorly between the base of the C7 transverse process and the neck of the first rib, posterior to the vertebral artery, medial to the scalene muscle group, and inferior to the apex of the lung.^7^, ^8^ SG has a good regulatory effect on the autonomic nervous system, the endocrine system, and the immune system. Related treatments are based on the involvement of the SG in the development and persistence of these chronic pain symptoms, such as stellate ganglion block (SGB), pulsed radiofrequency (PRF) for SG, and continuous radiofrequency (CRF) for SG.

## 3 SGI METHODS

### 3.1 SGB

SGB has been widely used to treat many kinds of chronic pain that occur in the upper extremities, neck, head, and orofacial region. Over the years, with the development of technology and efforts to improve the safety of the treatment, the techniques for SGB have evolved from the use of the standard blind technique, to fluoroscopy, computed tomography (CT) guidance, and ultrasound (US)‐guided approach recently.^9^, ^10^ Although there is a lack of extensive clinical data confirming the advantages of US‐guided SGB, US can better visualize peripheral nerves and tissue structures, reduce the risk of puncture, dynamically visualize diffusion around the injection, and reduce the local anesthetic dose.^11^ Furthermore, compared to CT or X‐rays, with the use of US, exposure to radiation risks can be avoided for both healthcare providers and patients.^11^ As C7 is located closer to the pleura, increasing the risk of pneumothorax, clinical procedures for SGB are usually performed at the C6 level rather than the C7 level.^12^ Current methods for assessing the effect of SGB are noninvasive and easy to obtain mainly including clinical symptoms (ptosis, facial flushing, conjunctival flushing), body temperature changes, perfusion index, and the visual analog scale (VAS) score. However, regardless of the method used for SGB, associated complications have been reported.^13^ Goel et al.^13^ reviewed SGB‐related complications, and found that most complications occurred during or shortly after operation, and the adverse events were mainly due to disruption of local structures (blood vessels, nerves, pleura, etc.), the local anesthetic is mistakenly injected into the blood vessel, or an effect caused by autonomic imbalance due to SGB.

Lee et al.^14^ recommended a volume of 2 ml of local anesthetic for US‐guided SGB as a safe and effective minimum dose. In their study, in total, there were 33 individuals with different types of chronic pain. They were divided into three groups according to the dose of mepivacaine (2, 3, or 4 ml). There were no significant differences among the three groups in terms of clinical outcomes (ptosis, conjunctival flushing) and contrast agent location. A recent study found that 4 ml of 1% lidocaine can exert similar clinical effects as larger volumes (6 and 8 ml) in US‐guided SGB.^15^ Both the above studies confirmed that US‐guided SGB was safer and involved a lower dose of local anesthetics compared with other interventional methods. However, there is still a lack of comparative experiments on dosages less than 2 ml and the degree of sympathetic block based on the perfusion index, which reflects the sympatholytic effects of SGB better than temperature.^14^, ^16^

### 3.2 PRF of SG

SGB is a useful tool for diagnosis and treatment, but the effects of local anesthetics last only for a few hours. SG PRF may extend these effects. PRF was first proposed for the treatment of pain by Sluijter in 1997.^17^ Then, it became a new tool in pain management. In PRF, also known as nondestructive radiofrequency, the intermittent pulse current emitted by the radiofrequency instrument is transmitted to the nerve in front of the needle vertically. PRF involves administration of a high voltage around the nerve tissue by intermittent radiofrequency current, and the temperature of the tissue is diffused during the time between the shots. This can diffuse the temperature generated so that the electrode temperature is typically below 42°C. Besides, this temperature will not damage the nerves, thereby avoiding complications such as excessive insomnia, pain, and movement disorders. In addition, the size of the lesion can be controlled by adjusting the treatment temperature, treatment time, and electrode size. In clinical trials, PRF has been proven to be effective for the treatment of many types of chronic refractory pain. In addition, it can be safely used where motor functions have to be retained.^18^

### 3.3 SG lesion with CRF of SG

CRF, also known as standard radiofrequency or radiofrequency thermal coagulation, uses high‐frequency current to produce high‐temperature effects to achieve tissue damage. Based on the treatment site, electrical impedance measurement, electrical stimulation response, and patient tolerance, the temperature can be set to 60–95°C, and the duration of thermocoagulation is usually 60–180 s. It is generally believed that if the temperature is set below 75°C, CRF thermocoagulation will not damage the motor nerve fibers. CRF of SG is usually performed after CT‐ or US‐guided localization to C7‐T1.^19^, ^20^ Besides, CRF of SG could lead to a longer duration and lower incidence of phenol sympathetic neuralgia as compared to phenol sympathetic blocks.^21^, ^22^

### 3.4 Noninvasive SGI

Noninvasive SGI refers to the stimulation of the projection area of the SG on the surface of the body by physical methods, such as US, transcutaneous electrical nerve stimulation, light irradiation using low‐level laser therapy, linearly polarized near‐infrared light irradiation (LPNIR), and xenon lamps, which can produce effects similar to nerve block. Current reports of noninvasive SGI therapy for chronic pain include complex regional pain syndrome (CRPS), fibromyalgia, tongue pain, burning mouth syndrome, and chronic pain due to other conditions.^23^, ^24^, ^25^, ^26^, ^27^ Compared with SGB, these treatments are noninvasive and free from potential complications such as local anesthetic allergy or intoxication, puncture injury, and other adverse events due to repeated treatments. It has been found that irradiation near the SG region could effectively relieve neuropathic pain, and this pain was related to the heart rate variability,^28^ indicating that the sympathetic nervous system had been inhibited. Therefore, for patients with hemophilia and other patients with a higher bleeding tendency, noninvasive SGB can avoid the bleeding caused by invasive treatment and become a suitable choice.^28^ Although multiple irradiations are also required to maintain the effect of noninvasive treatment, noninvasive SGB may be a suitable choice for patients with contraindications to SGB, such as hemophilia and other patients with a higher bleeding tendency.

## 4 SGI INDICATIONS AND EFFECTS

### 4.1 Postherpetic neuralgia

Postherpetic neuralgia (PHN) is the most common complication of shingles, often accompanied by hyperalgesia and hypersensitivity, that severely affects the quality of life.^29^ Due to the high sensitivity of the face and the extremities to pain, these areas are at high risk for PHN.^30^ The pathogenesis of PHN is complex, and there is no effective treatment.^31^, ^32^, ^33^ Besides, studies have confirmed that early SGB can not only effectively relieve herpes acute pain but also reduce the incidence of PHN.^34^ However, due to the short maintenance time after a single SGB treatment, multiple injections are generally required for maintenance treatment, which increases the risk of injection complications. Repeated treatments can lead to distress, poor compliance, increased financial burden, and poor quality of life. As an alternative to traditional SGB, noninvasive treatment of PHN using low‐intensity laser or percutaneous light irradiation with LPNIR near the SG area did not lead to complications or side effects.^28^ Recently, in a retrospective study with 84 patients with PHN in the face or upper limbs, a single SG PRF (42°C for 300 s) significantly alleviated pain and improved quality of life compared to that of patients receiving SGB.^35^

### 4.2 Complex regional pain syndrome

CRPS is a localized pain syndrome associated with abnormal sensory, motor, autonomic, skeletal, and skin changes, usually following trauma or surgery. Autonomic nervous system dysfunction is considered to be an important factor in the development and persistence of CRPS.^36^ CRPS is often associated with autonomic dysfunction or dystrophy, and is accompanied by symptoms of sympathetic activity, such as changes in skin temperature and color, edema, and sweating.^37^ Nearly half of patients with CRPS reported greater than 50% pain reduction after SGB treatment, and 17% reported at least 30% pain reduction.^38^ Besides, in poststroke CRPS and in CRPS patients with failure of previous treatment, pain reduced by up to 74% and 83%, respectively.^36^, ^39^ This range of pain reduction can likely be explained by different inclusion criteria and differences in the study design. Yet, all reports confirm that SGB can reduce pain in CRPS. In a cohort of 287 CRPS patients receiving SGB, more than half of the patients were moderately satisfied with the treatment outcome. However, to maintain the treatment effect, more than half of the patients also received more than one SGB treatment.^40^ Therefore, the duration of the effect of SGB is usually temporary. To prolong the treatment effect and reduce possible complications caused by repeated treatments, multiple studies have confirmed that PRF is feasible in CRPS patients.^19^, ^41^ In a clinical trial, 91.7% of CRPS patients with positive SGB reactions experienced at least moderate improvement after SG PRF treatment.^41^ Also, this reduction in symptoms was maintained for a mean of 31.41 ± 26.07 days after PRF.^41^ In another retrospective study of chronic refractory type I CRPS of the upper limb, the treatment outcomes of CT‐guided SG radiofrequency neurolysis were better than those with SGB (67.6% vs. 21.2%), and pain relief lasted up to 2 years.^42^ Besides, successful pain relief for 14 months has been reported in CRPS II patients received one SG PRF.^19^

### 4.3 Facial pain

SGB has been reported to be effective in relieving different types of facial pain, including orofacial cancer pain, atypical facial pain, neuropathic pain after simple tooth extraction, and postoperative eye pain.^43^, ^44^, ^45^, ^46^, ^47^, ^48^ Darabad et al.^43^ performed SGB in three patients with cancer‐related facial pain. At a 3‐month follow‐up after SGB, they observed at least a 40% reduction in pain. Besides, in a case of carcinoma buccal mucosa with chronic facial pain, PRF of SG resulted in up to 75% pain relief at the second‐week follow‐up and the sixth‐week follow‐up.^44^ Atypical facial pain is also known as persistent idiopathic facial pain, and the pathogenesis may be related to autonomic dysfunction, psychological factors, central sensitization, demyelination, and axonal dysfunction.^49^ It has been reported that SGB can be used to effectively treat refractory atypical facial pain^45^, ^46^ and/or burning mouth syndrome.^50^ Performing the SGI in the early stages of various oral and facial pain disorders may result in a greater reduction in the severity of pain.^51^

### 4.4 Fibromyalgia syndrome

Fibromyalgia syndrome (FMS) is a multisystem illness characterized by chronic, widespread musculoskeletal pain and associated fatigue, sleep disturbances, and other cognitive and somatic symptoms.^52^ Studies have found that many FMS patients have symptoms of sympathetic nervous system dysfunction such as sleep disturbance, body stiffness, fatigue, cognitive dysfunction, and so forth.^53^, ^54^ Bengtsson^55^ published the first study of SGB, a controlled therapeutic trial, in the treatment of FMS in 1988. They reported, in contrast to the group with a sham injection, in SGB group, the rest in pain and the number of trigger and tender points in patients with primary fibromyalgia markedly reduced in response to SGB.^55^ Recently, in Nakajima et al.'s^23^ study, it was found that LPNIR (0.38–1.1 μm) over the SG region led to a decrease in the VAS score in patients with fibromyalgia and higher scores on the Japanese version of the fibromyalgia impact questionnaire (J‐FIQ), but it was a single irradiation combined with drug therapy; the evaluation of the long‐term effects of LPNIR alone was impossible. Therefore, SGI can be an option for the treatment of FMS, but more clinical evaluations still need to be carried out on the clinical effect and safety.

### 4.5 Other pain

SGI not only treats pain associated with sympathetic nerve maintenance also but other types of chronic pain, such as postmastectomy pain syndrome (PMPS) and chronic nerve pain.^56^, ^57^ In a randomized‐controlled study of thermal versus supervoltage PRF of SG in PMPS, SG thermal radiofrequency significantly reduced postmastectomy pain in 80 enrolled patients.^56^ They also showed functional improvement, and used fewer rescue pain killers compared with the PRF group in the 6‐month follow‐up period.^56^ Recently, the application of PRF of SG  was reported in a 62‐year‐old woman with chronic nerve pain due to pleural malignant tumor encasing the cervical nerve roots.^57^ It was found that the VAS score was 0/10 after SG PRF and at the 3‐year follow‐up.^57^

## 5 ANALGESIC MECHANISMS OF SGI

The process by which SGI induces analgesia is multifactorial and likely encompasses peripheral and central mechanisms. Studies have confirmed that after peripheral nerve injury, innervation is formed between the dorsal root ganglion (DRG) and the terminals of sympathetic efferent fibers, which means that the activity of sympathetic efferent fibers can cause abnormal activities and responses of peripheral afferent fibers.

In an animal study, after peripheral nerve injury, cutaneous C‐fiber nociceptors expressing alpha‐2 adrenergic receptors were found to be sensitive to sympathetic nerve stimulation and norepinephrine (NE).^58^ Perhaps one of the mechanisms of sympathetically maintained pain syndrome is the sympathetic sprouting at the DRG, where axonal terminals of sympathetic nerves proliferate and invade sensory neurons to form basket‐like structures.^59^ Besides, these neurons can be repeatedly activated by sympathetic stimulation.^59^ In addition, Zhang et al.^60^ discovered a unique form of abnormal spontaneous activity following peripheral nerve injury: synchronous and sporadic firing of adjacent clusters of DRG neurons. Furthermore, they demonstrated that cluster firing is triggered by sympathetic activity and identified NE as a key neurotransmitter regulating this unique firing.^60^ As one of the important sympathetic ganglions, SGI intervention may relieve pain associated with sympathetic maintenance by inhibiting sprouting and new nerve growth through sympathetic modulation.

An increase in NE had been shown to be associated with posttraumatic stress disorder (PTSD), CRPS, and hot flashes.^61^, ^62^, ^63^ In rabbits, the nociceptive responses induced by formalin injection (an inflammatory neuropathic pain model) decreased with the application of SGB.^64^ Importantly, the levels of substance P in the spinal cord and plasma catecholamine in the formalin + SGB group were lower than those in the formalin group.^64^ By using the pseudorabies virus, it was found that a stimulatory signal originating from SG went “upstream” to the hypothalamus, amygdala, and hippocampus.^65^ In turn, these areas had been shown to have reciprocal innervation with locus coeruleus, which was the main synthesis site of NE in the brain.^66^ This suggests that the effects of SGB effects may be mediated by lower levels of NE by modulating sympathetic nervous system activity. SGB can block the reflex pathway of the spinal cord, reduce the excitability and sensitivity of sympathetic nerves, induce the disappearance of local vasoconstriction, increase regional blood flow, improve ischemia and hypoxia, eliminate NE, substance P, and other mediators,^45^, ^64^ regulate the early inflammation responses, inhibit the proinflammatory cytokines interleukin 1β (IL‐1β), tumor necrosis factor‐alpha, and IL‐6, and promote nerve repair.^67^

SGB with 0.2 ml 0.25% bupivacaine can significantly reduce brain electrical activity in rats.^68^ Similar results had also been observed in humans, where SGB with lidocaine led to significantly decreased bispectral index (BIS) values and Observer's Assessment of the Alertness/Sedation scores as compared to the baseline.^69^ These results suggested that SGB can induce a sedative effect in humans. Also, these results can be explained by the reduction of NE levels in the brain.^70^ The analgesic mechanism of PRF is not very clear, but temperature changes are not responsible for pain relief. Another hypothesized mechanism reduction of the release of substance P to nociceptive stimuli, thereby leading to a reduction in nociceptive behavior and a reduction in hyperalgesia. Recently, studies have found that SGB can effectively relieve symptoms in patients with anxiety disorders and PTSD.^71^ Therefore, the sedative and antidepressant effects of SGB may alleviate symptoms of chronic pain. However, these experiments did not directly address pain, so further research is needed.

## 6 CONCLUSION

**Figure 1:**
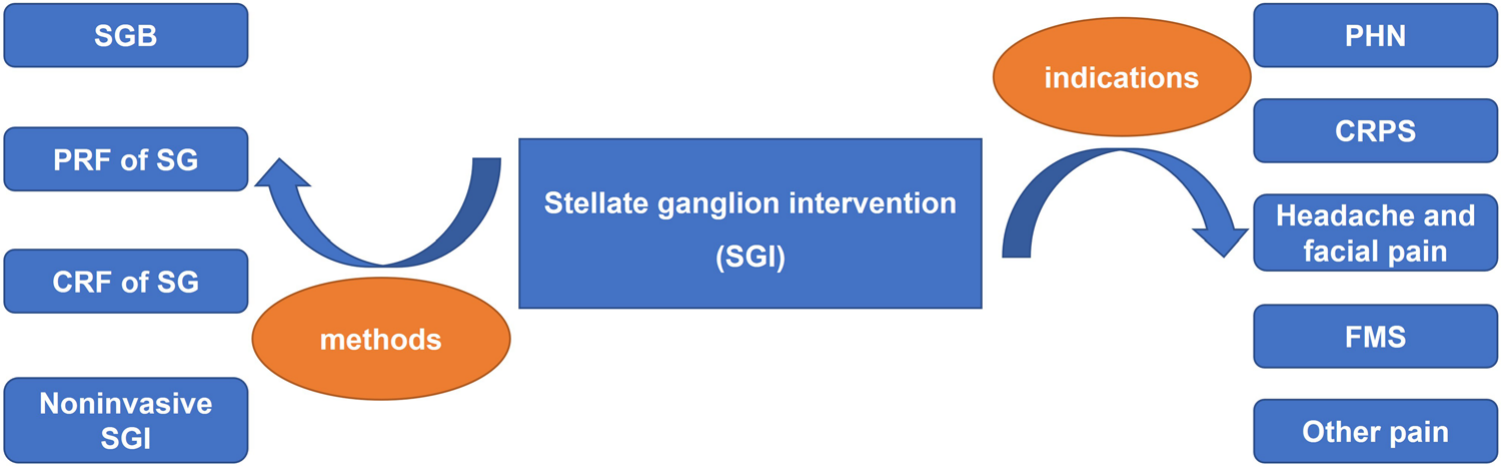
Methods and indications of stellate ganglion intervention (SGI). CRF, continuous radiofrequency; CRPS, complex regional pain syndrome; FMS, fibromyalgia syndrome; SG, stellate ganglion; SGB, stellate ganglion block; PHN, postherpetic neuralgia; PRF, pulsed radiofrequency. [Color figure can be viewed at wileyonlinelibrary.com]

The interventions of SG have been applied in many kinds of chronic pain conditions (Figure 1), such as CRPS, PHN, PTSD, headache, and facial pain (Table 1). In addition, compared with SGB, the radiofrequency treatment of SG can increase the effective remission period without severe complications.^19^, ^41^ Still, more studies, including prospective randomized‐controlled trials, are necessary to evaluate the effects and safety of SGI, especially SG PRF and SG CRF. The analgesic mechanism of SGI in the treatment of chronic pain is still unclear, but it may be through sympathetic modulation that inhibits sprouting and new nerve growth to relieve pain associated with maintaining sympathetic nerve.

**Table 1:** Summary of stellate ganglion intervention (SGI) therapy for chronic pain.

Disease	SGI type	Study type	Treatment protocol
PHN	SGB^34^	Randomized‐controlled trial	6 ml 0.125% bupivacaine and 8 mg dexamethasone.
	LPNIR^28^	Prospective double‐blind, randomized study	1800 mW; irradiation for 2 s, followed by a 4 s pause; irradiation duration of 10 min.
	PRF^35^	Randomized‐controlled trial	42°C for 300 s (pulse width: 20 ms, frequency: 2 Hz).
CRPS	SGB^36^, ^38^, ^40^	Descriptive study	A volume of 15 ml of equal parts 0.5% bupivacaine and 1% prilocaine–hydrochloride, three times, with an interval of 1 week between treatments.
		Retrospective observational study	5 ml of 0.5% bupivacaine; 5 ml of 1% ropivacaine; 3 ml of 1% ropivacaine; and 2 ml of 1% ropivacaine + 30 µg of clonidine in 1 ml saline, resulting in a volume of 3 ml.
		Descriptive study	5 ml of 0.3% bupivacaine and 4 mg of dexamethasone.
	PRF^19^, ^41^	Case report	42°C (2 Hz, 45 V), two cycles of 120 s.
		Retrospective observational study	PRF was performed for 420 s at 42°C on the C6‐ and C7‐level sympathetic chain.
	RFN^42^	Retrospective observational study	Place needles sequentially at the C7 and TI levels and perform three 60‐s RFN cycles at each level in injury mode at 70, 80, and 90°C.
	LPNIR^24^	Descriptive study	Linearly polarized 0.6–1.6 mm light (0.92 W, 88.3 J).
Facial pain	SGB^15^, ^43^, ^45^, ^46^	Randomized‐controlled trial	4, 6, or 8 ml of 1.0% lidocaine.
		Case series	10 ml of 0.25% bupivacaine.
		Case report	6 ml of 1.5% lidocaine, 2–3 times a week, 12 times in total.
		Case report	0.25% bupivacaine (5 ml) mixed with 8 mg of dexamethasone.
	PRF^35^, ^44^	Randomized‐controlled trial	42°C for 300 s (pulse width: 20 ms, frequency: 2 Hz), two cycles.
		Case report	42°C for 60 s, twice.
	LPNIR^26^	Case series	Power: 5.0 W, Pulse width: 3 ms, interpulse period: 7 ms, duration: 3 min, once a week for 10 weeks.
FMS	SGB^55^	Randomized‐controlled trials	15 ml of 0.25% bupivacaine.
	LPNIR^23^	Descriptive study	Bilateral xenon light irradiation (0.38–1.1 μm) around the SG.
PMPS	TRF^57^	Prospective randomized trial	Perform nerve release for 60 s at 80°C, then repeat twice after needle tip rotation.

Abbreviations: CRPS, complex regional pain syndrome; FMS, fibromyalgia syndrome; LPNIR, linearly polarized near‐infrared light irradiation; PHN, postherpetic neuralgia; PMPS, postmastectomy pain syndrome; PRF, pulsed radiofrequency; RFN, radiofrequency neurolysis; SGB, low‐level laser therapy; TRF, thermal radiofrequency.

## AUTHOR CONTRIBUTIONS

Qingyang Luo and Song Cao contributed to the main conception of this review, which led to the submission and resource collection. Song Wen, Xinran Tan, and Xi Yi contributed toward organizing ideas and revising the article. Song Cao reviewed and edited the paper.

## CONFLICT OF INTEREST

The authors declare no conflict of interest.

## ETHICS STATEMENT

There are no possible animal or medical ethical issues for this review article.

## Data Availability

The data reported in this study are available from the lead contact on request.
